# Nursing performance in COVID-19 and non-COVID-19 units: Implications for occupational health

**DOI:** 10.1590/1518-8345.6215.3741

**Published:** 2023-01-30

**Authors:** Larissa Fonseca Ampos, Luciana Olino, Ana Maria Müller de Magalhães, Juliana Petri Tavares, Tânia Solange Bosi de Souza Magnago, Daiane Dal Pai

**Affiliations:** 1 Universidade Federal do Rio Grande do Sul, Porto Alegre, RS, Brazil; 2 Scholarship holder at the Coordenação de Aperfeiçoamento de Pessoal de Nível Superior (CAPES), Brazil; 3 Universidade Federal do Rio Grande do Sul, Departamento de Enfermagem Médico-Cirúrgica, Porto Alegre, RS, Brazil; 4 Universidade Federal de Santa Maria, Departamento de Enfermagem, Santa Maria, RS, Brazil

**Keywords:** Nursing, COVID-19, Occupational Health, Mental Health, Occupational Exposure, Working Conditions, Enfermagem, COVID-19, Saúde do Trabalhador, Saúde Mental, Exposição Ocupacional, Condições de Trabalho, Enfermería, COVID-19, Salud Laboral, Salud Mental, Exposición Ocupacional, Condiciones de Trabajo

## Abstract

**Objective:**

to analyze the implications of the pandemic on the Nursing team’s occupational health according to its performance in COVID-19 and non-COVID-19 units.

**Method:**

a multicenter and mixed-methods study, with a sequential explanatory strategy. A total of 845 professionals took part in the first stage, answering an electronic form which contained sociodemographic and work-related variables, as well as about the pandemic and their health, in addition to the Self-Reporting Questionnaire. 19 professionals were interviewed in the second stage. The quantitative data were submitted to statistical analysis and the qualitative ones to thematic content analysis, with integration by connection.

**Results:**

the pandemic exerted impacts on the professionals’ health, both in the COVID-19 and non-COVID-19 areas. However, composition of the teams presented different characteristics between the areas, as well as the risk perceptions and the work demands.

**Conclusion:**

the professionals working in areas COVID-19 and non-COVID-19 areas are equally affected, although with different work exposure regarding the requirements at work in the COVID-19 units and the fear of contamination in non-COVID-19 units.

## Resumo

**Objetivo:**

analisar as implicações da pandemia sobre a saúde ocupacional da equipe de enfermagem conforme a atuação em unidades dedicadas e não dedicadas à COVID-19.

**Método:**

estudo multicêntrico, de método misto, estratégia explanatória sequencial. Participaram 845 profissionais na primeira etapa, respondendo a um formulário eletrônico contendo variáveis sociodemográficas, laborais, sobre a pandemia e sua saúde e o *Self-Reporting Questionnaire*. Na segunda etapa, 19 profissionais foram entrevistados. Os dados quantitativos foram submetidos à estatística analítica e os qualitativos à análise temática de conteúdo, sendo integrados por conexão**.**

**Resultados:**

os impactos da pandemia sobre a saúde dos profissionais ocorreram tanto nas áreas dedicadas como nas não dedicadas à COVID-19. Contudo, a composição das equipes apresentou características distintas entre as áreas, bem como as percepções de risco e das exigências no trabalho.

**Conclusão:**

os profissionais que atuam em áreas dedicadas e não dedicadas à COVID-19 estão igualmente adoecidos, mas com exposição laboral distinta quanto às exigências no trabalho nas unidades dedicadas e o medo da contaminação nas unidades não dedicadas.

## Resumen

**Objetivo:**

analizar las consecuencias que tuvo la pandemia en la salud ocupacional de los enfermeros según si se desempeñaban en unidades dedicadas y no dedicadas al COVID-19.

**Método:**

estudio multicéntrico, método mixto, estrategia explicativa secuencial. En la primera etapa participaron en total 845 profesionales que respondieron un formulario electrónico que contenía variables sociodemográficas, ocupacionales, sobre la pandemia y la salud y el *Self-Reporting Questionnaire*. En la segunda etapa, fueron entrevistados 19 profesionales. Los datos cuantitativos fueron sometidos a estadística analítica y los datos cualitativos al análisis de contenido temático, posteriormente, ambos fueron integrados por conexión.

**Resultados:**

los impactos de la pandemia en la salud de los profesionales se produjeron tanto en las áreas dedicadas como en las no dedicadas al COVID-19. Sin embargo, la composición de los equipos presentó distintas características entre las áreas, así como también diferentes percepciones de riesgo y exigencias en el trabajo.

**Conclusión:**

los profesionales que trabajan en áreas dedicadas y no dedicadas al COVID-19 se enfermaron por igual, pero tuvieron diferente exposición ocupacional debido a las exigencias que presentaba el trabajo en las unidades dedicadas y al miedo al contagio en las unidades no dedicadas.

**Highlights**

(1) The COVID-19 pandemic exerted an impact on Nursing workers’ health.

(2) Composition of the teams differed in the COVID-19 and non-COVID-19 units.

(3) Working in COVID-19 units intensified work pace and complexity.

(4) Working in non-COVID-19 units increased the fear of contracting the infection.

(5) The suspicion of Minor Psychological Disorders is high, with no difference regarding the units

## Introduction

Among the challenges imposed by the coronavirus pandemic, health services faced rapid and important adaptations given the impact of morbidity and mortality due to COVID-19 on the population, which was aggravated by the global scarcity of inputs to protect health professionals^1^ ^-^ ^2^. Therefore, the Nursing team, which works in the most varied health services, stands out with regard to exposure to illness risk and vulnerability, either because its work requires physical and close contact with the patients or even in relation to care provided in long working hours^3^ ^-^ ^4^.

In order to control spread of the infection in the health services, hospital institutions have designated units devoted to the care of patients infected with the coronavirus, seeking to keep them separate from other hospitalizations due to causes other than COVID-19^5^. Considering performance in these units, the intensity of the requirements related to the adaptations of the flows and processes for the assistance to the victims of the disease stands out, including clinical management of the symptoms and elaboration of work protocols, as well as the need to use Personal Protective Equipment (PPE) for extended periods of time and for paramentation and de-paramentation training sessions since, in the COVID-19 units, high exposure to aerosols increases contact with the virus^5^ ^-^ ^8^.

The increased exposure risk in these workers, added to the requirement of technical improvement and adaptations in the schedules, routines and work protocols, increased the professionals’ workload^8^ ^-^ ^10^, also added to the social restrictions imposed by the pandemic on domestic life and cessation of essential leisure activities in the mediation of pressures and work-related stress^11^ ^-^ ^12^.

In this context, workers working in non-COVID-19 units have not undergone the same organizational changes, which could immediately represent a lower impact on them. However, as they do not enjoy the same precautions offered in the COVID-19 units, they faced insecurity due to the unidentified presence of the virus, either due to absence/ineffectiveness of tests to detect the infection, to asymptomatic cases, to nonspecific symptoms or to symptoms that become suspicious during hospitalization^13^.

These situations caused by the COVID-19 pandemic favor tiring and detrimental work environments and relationships for Nursing professionals, which become even more susceptible to developing stress, wear out, insomnia, anxiety and depression, among other symptoms that cause distress, illness and leaves of absence, as already shown in studies conducted with Indian and Chinese workers through Minor Psychological Disorders (MPD)^14^ ^-^ ^16^.

MPD consist of depression, anxiety, fatigue, irritability, insomnia and memory and concentration deficit^17^ symptoms and have been identified in Brazilian Nursing and in other countries^18^ ^-^ ^20^. In addition to the harms to workers’ health, MPD can negatively interfere on the work process and on patient safety.

In the international context, there are studies^15^ ^,^ ^21^ ^-^ ^22^ pointing out to the greater vulnerability of the professionals who worked directly with patients infected with the coronavirus, although another does not corroborate this result^23^. In the Brazilian context, no studies were found that compared the impacts of coping with the pandemic between areas of professional activity devoted and not devoted to the care of coronavirus-infected patients.

The importance of this study is justified by the need to gather subsidies to more assertively direct the efforts in the protection, prevention and promotion of Nursing professionals’ health, who have been protagonists at this moment of facing the pandemic. Although workers in COVID-19 units were permanently exposed to the virus, this study hypothesized that the impact of the pandemic on the professionals’ health affected both workers in COVIC-19 and non-COVID-19 units, as all faced new and/or increased demands due to the pandemic context, as well as exposure to the virus even before confirmation of the diagnosis. Therefore, the objective was to analyze the implications of the pandemic on the Nursing team’s occupational health according to its performance in COVID-19 and non-COVID-19 units. 

## Method

### Study design

This is a multicenter study with a mixed-methods approach and a sequential explanatory strategy. This strategy uses greater weight assignment in collection of quantitative data (QUAN) to elaborate the qualitative stage, whose weight assignment is lower (qual), both stages being combined by connection^24^. A cross-sectional designed was used in the quantitative stage and the qualitative stage was descriptive, with content analysis methodological guidance^25^. This study was guided by the STROBE (Strengthening the Reporting of Observational Studies Epidemiology) guideline, which is used to describe observational studies^26^.

### Data collection locus (city, state acronym and country)

The study was conducted in units intended and not intended to the care of COVID-19 patients, from four hospital institutions, located in the central and eastern regions of Rio Grande do Sul (RS) state, in Brazil. The hospitals are tertiary-level or references in care by the Unified Health System and are referred to as HA, HB, HC and HD, with 784, 237, 919 and 403 beds, respectively.

### Period

The first stage (QUAN) was conducted from August to October 2020 and the second, from January to May 2021.

### Population

The study population consisted of 2,962 Nursing professionals from hospital HA, 707 from HB, 2,278 from HC and 952 from HD, totaling 6,899 Nursing workers (nurses, technicians and nursing assistants).

### Selection criteria

In the “QUAN” stage, all the workers from the four institutions were invited by means of institutional email messages, with inclusion of those who answered the electronic form. A sample of these respondents was included in the “qual” stage selected from the statements written in the open question, providing contact to talk more about the subject matter. The professionals excluded were those that were away from their work functions during the data collection period and who refused to be interviewed.

### Sample definition

The “QUAN” stage consisted of a sample of 845 participants from the Nursing team, selected by convenience, exceeding the minimum number (534) statistically estimated with the aid of the statistical PSS Health software (Power and Sample Size for Health Researchers)^27^, with a 96% confidence level and a 4% estimate of absolute error, assuming 33.7% prevalence^28^. The “qual” stage sample was intentionally obtained among the 353 individuals who answered the open question and defined at 19 participants through data saturation. Intentionality of the selection of interviewees occurred by choosing the best informants, through identification the answers that revealed interest in discussing their work experiences during the pandemic.

### Study variables

The sociodemographic data were collected through dichotomous categorical variables such as sex (female or male), race/skin color (white or brown/black/others), marital status (single/without a partner or married/with a partner) and numerical variables such as number of children. The work-related data were categorical polytomous variables such as institution (HA, HB, HC or HD), work shift (day, night or no fixed turn/relief staff), dichotomous variables such as position (nurse or nursing technician/assistant), employment contract [Consolidation of Labor Laws (CLT-*Consolidação das Leis do Trabalho*)]/statutory or temporary] and if they had another contract (yes or no). The numerical variables were “time working in the institution”, “time working in the profession” and “time working in the unit” (in years).

MPD suspicion, as well as data on life and health habits related to the pandemic (has a chronic disease, practices physical activity, increased alcohol consumption, works in a unit devoted to infected patients, assisted infected patients, started using medication, had a medical leave, due to suspected or confirmed COVID-19, belongs to the risk group, lives with people who belong to the risk group) were obtained through dichotomous categorical variables (yes or no), in addition to the “time away” numerical variable (in days). A five-point Likert scale was used for the following variables: sleep quality (from poor: “1” to excellent: “5”), increased level of demand at work (from nothing changed: “1” to intensely changed: “5”), fear felt in the face of exposure to being contaminated at work (from not afraid: “1” to very much afraid: “5”) and impact on physical health (from no impact: “1” to intense impact: “5”).

The study considered the sectors that devoted their physical area and care process exclusively to patients infected by the coronavirus as “COVID-19 units”. The other sectors were understood as “non-COVID-19 units”, intended to care for other causes (such as outpatient services and hospitalizations of any specialties) and/or that were adapted to carry out eventual care of patients infected with the coronavirus, their permanence being temporary in the area (such as emergencies and areas for exams).

### Instruments used for data collection

For collecting quantitative data, an online form prepared by the authors of the manuscript and submitted to a pilot test was used, containing sociodemographic, work-related, pandemic and health questions, in addition to the Self-Reporting Questionnaire (SRQ-20) that investigates MPD. SRQ-20, validated in Brazil in 1986^29^, consists of 20 dichotomized questions (yes/no), divided into symptoms characteristic of depressive-anxious mood, somatic mood, decreased vital energy and depressive thoughts. Results equal to or higher than 7 were used as cutoff point to define suspected MPD, without resulting in diagnosis.

The open question available in the electronic form, to freely discuss their experiences during care performance in the pandemic, was used to guide the development of semi-structured questions that comprised the interview script of the second stage, as well as the results of the previous analysis of the data from the first stage of the study. The interview script, prepared by the authors of the study, consisted of six questions that dealt with daily work in the pandemic, organization of teams and processes, perceived changes, exposure to risk and impact on health.

### Data collection

Quantitative data collection was conducted by means of an electronic form (*Google Forms*), sent via institutional email, provided and authorized by the institutions. The qualitative stage was conducted by the researcher, first author of this manuscript, in the remote modality and through the *Google Meet* videocall platform, with recording of the interviews. Access to the participants who answered the interview was through the email address provided in form from the first stage. The interviews were scheduled according to the participants’ availability.

### Data treatment and analysis

The “QUAN” stage data were entered into an *Excel* spreadsheet and analyzed in SPSS, version 20. The categorical variables were presented as absolute and relative frequencies and the continuous ones, as central tendency and dispersion measures. The Chi-Square or Fisher’s Exact tests were employed for the association between the categorical variables, according to the cell’s frequency. The Shapiro-Wilk normality test resulted in the identification of the asymmetric distribution of the continuous variables, which had their analysis performed using the non-parametric Mann-Whitney test in the comparison of the groups. “Works in a unit devoted to infected patients” was the dependent variable of this study, which was crossed with the independent ones. The data with two-tailed “p”-values below 0.05, or with a 95% confidence interval, were considered as statistically significant differences.

In the “qual” stage, the data were transcribed and submitted to thematic content analysis^29^, permeating the pre-analysis phases, represented by intense floating reading until impregnation of the material, where the first impressions that emerge in the researcher are allowed to flow; exploration of the material, to understand the findings, where elaboration of the categories begins, reducing the material to words and speeches and inference and interpretation of the results, the final phase of the analysis.

After the quantitative and qualitative analyses, a joint data analysis was performed, combined from the connection, so that the results could be integrated, improving and expanding understanding of the theme. Joint-display was used to allow for better data visualization^30^, favoring the emergence of new ideas^31^.

### Ethical aspects

This study was approved by the National Commission on Research Ethics under opinion number 4,152,027 and CAAE record 33105820200000008. The ethical principles were followed as provided in Resolution No. 466 of December 12^th^, 2012, by the National Health Council of the Ministry of Health, as well as the norms applicable for research in Human and Social Sciences provided in Resolution No. 510/16. The Free and Informed Consent From was sent together with the online form, informing about both stages. In the interviews, the professionals’ names were substituted by the acronyms NT for Nursing Technician/Assistant and NUR for Nurse.

## Results

The study first stage included 845 Nursing team professionals from all four hospitals: 155 participants from HA, 90 from HB, 367 from HC and 233 from HD. The participants included 470 (55.6%) nursing technicians/assistants and 375 (44.4%) nurses.

There was predominance of the female sex (84.9%), with a median age of 41 (36-48) years old and self-declared as white race/skin color (83.1%) and 625 (74%) were married or had partners. Regarding the participants’ health, 580 (68.6%) answered that they were not practicing physical activity, 200 (23.7%) increased alcohol consumption in the pandemic, 205 (24.3%) started using medication in this period and the prevalence of suspected MPD was 49.3% (417).

Regarding the work-related data, 762 (90.2) participants were statutory or CLT-contracted, 112 (13.3%) had another employment contract, 566 (67%) were from the day shift and 64 (7.6) had a management position. The sample consisted of 155 professionals working in COVID-19 units and of 690 from non-COVID-19 units. However, regarding the experience of the care provided to infected patients, it was found that 575 (83.3%) had these experiences working in non-COVID-19 units.

Table 1 shows the distribution of participants according to their sociodemographic and work-related characteristics according to the work unit in coping with the COVID-19 pandemic. Table 2 defines the changes in life and health habits and in the work context due to the pandemic.

**Table 1:** Distribution of the Nursing professionals from all four institutions (n=845) according to COVID-19 or non-COVID-19 work unit. Rio Grande do Sul, RS, Brazil, 2020-2021

Variables	COVID-19 unit		p*
	Yes (n=155)	No (n=690)	
*Sex*			0.004
Female	120 (77.4)	597 (86.5)	
Male	35 (22.6)	93 (13.5)	
*Skin color*			0.015
White	119 (76.8)	583 (84.5)	
Brown, black and others	36 (23.2)	107 (15.5)	
*Has some chronic disease*			<0.001
No	106 (68.4)	359 (52.0)	
Yes	49 (31.6)	331 (48.0)	
Sleep quality	2.9 ± 1^†^	3 ± 1^†^	0.132
*Marital status*			0.739
Single/No partner	42 (27.1)	178 (25.7)	
Married/With partner	113 (72.9)	512 (74.2)	
Number of children	1.03 ± 0.968^†^	1 ± 0.915^†^	0.232
*Institution*			<0.001
HA	20 (12.9)	135 (19.6)	
HB	12 (7.7)	78 (11.3)	
HC	110 (71.0)	257 (37.2)	
HD	13 (8.4)	220 (31.9)	
*Shift*			<0.001
Day	85 (54.8)	481 (69.7)	
Night	62 (40.0)	179 (25.9)	
No fixed shift/Relief staff	8 (5.1)	30 (4.4)	
*Employment contract*			<0.001
CLT^‡^/Statutory	106 (68.4)	656 (95.1)	
Temporary	49 (31.6)	34 (4.9)	
*Has another employment contract*			<0.001
No	116 (74.8)	617 (89.4)	
Yes	39 (25.2)	73 (10.6)	
Time working in the profession in years	13.7 ± 7.4^†^	16.2 ± 8.9^†^	0.002
Time working in the institution in years	6.73 ± 7.7^†^	8.6 ± 8.4^†^	<0.001
Time working in the unit	1.83 ± 5.4^†^	5.83 ± 6.8^†^	<0.001

* p-value; ^†^Mean and Standard Deviation; ^‡^ *Consolidação das Leis do Trabalho* (Consolidation of Labor Laws). Note: Considering the asymmetrical distribution of the continuous variables, the Mann-Whitney test was employed. However, it was decided to present the mean values and standard deviations in order to ease interpretation of the findings

**Table 2:** Repercussions of the pandemic according to COVID-19 or non-COVID-19 work unit (n=845). Rio Grande Sul, RS, Brazil, 2020-2021

Variables	COVID-19 unit		p*
	Yes (n=155)	No (n=690)	
*Increase in alcohol consumption during the pandemic*			0.266
No	113 (72.9)	532 (77.1)	
Yes	42 (27.1)	158 (22.9)	
*Practice of physical activity during the pandemic*			0.283
No	112 (72.2)	468 (67.8)	
Yes	43 (27.8)	222 (32.2)	
*Risk group for COVID-19*			<0.001
No	135 (87.1)	496 (71.9)	
Yes	20 (12.9)	194 (28.1)	
*Lives with people belonging to the risk group for COVID-19*			0.219
No	92 (59.4)	372 (53.9)	
Yes	63 (40.6)	318 (46.1)	
Fear towards risk exposure	3.48 ± 1.2^†^	3.70 ± 1.2^†^	0.033
*Started using medication during the pandemic*			0.340
No	122 (78.7)	518 (75.1)	
Yes	33 (21.3)	172 (24.9)	
*Distancing from work due to health reasons during the pandemic*			0.218
No	96 (61.9)	390 (56.5)	
Yes	59 (38.1)	300 (43.5)	
*Distancing from work due to suspected COVID-19*			0.351
No	86 (55.5)	411 (59.6)	
Yes	69 (44.5)	279 (40.4)	
*Distancing from work due to COVID-19 diagnosis*			0.221
No	131 (84.5)	608 (88.1)	
Yes	24 (15.5)	82 (11.9)	
Days away during the pandemic	10.5 ± 9.4^†^	11.8 ± 13.1^†^	0.549
Increase in the demand level during the pandemic (pace and complexity)	4.5 ± 0.7^†^	4.0 ± 1.1^†^	<0.001
Impact on physical health	3.5 ± 1.1^†^	3.6 ± 1.1^†^	0.447

* p-value; ^†^Mean and Standard Deviation. Note: Considering the asymmetrical distribution of the continuous variables, the Mann-Whitney test was employed. However, it was decided to present the mean values and standard deviations in order to ease interpretation of the findings.

**Figure 1:**
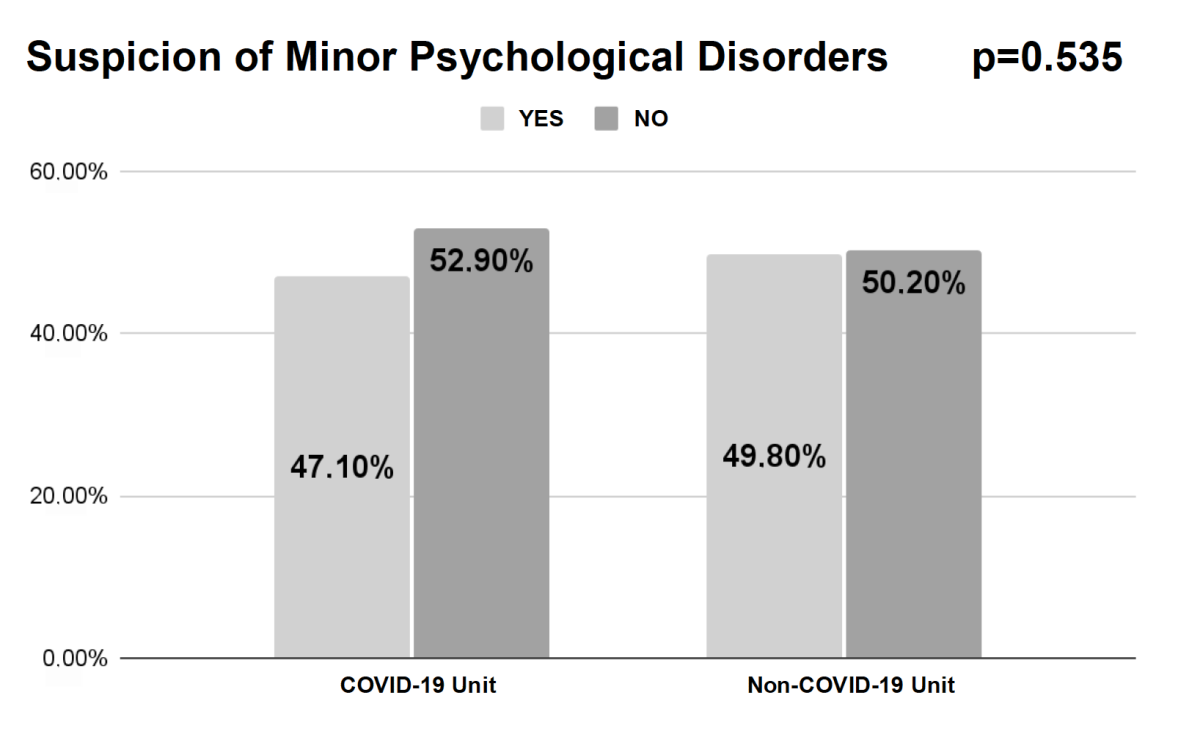
Prevalence of Minor Psychological Disorders in the COVID-19 (n=155) and non-COVID-19 (n=690) areas. Rio Grande do Sul, RS, Brazil, 2020-2021

Regarding the MPD, there were no statistically significant differences between the COVID-19 and non-COVID-19 areas. Figure 1 illustrates the high prevalence of suspected MPD in the Nursing professionals from both teams.

In the “qual” stage, 19 professionals were interviewed, of which 9 were nurses and 10 were nursing technicians, 10 of whom were from COVID-19 units and 9 from non-COVID-19 units and distributed across all four hospitals as follows: 5 in HA, 3 in HB, 5 in HC and 6 in HD. Three categories emerged from analyzing these interviews: (1) Restructuring the work teams; (2) Understanding risk and work demands; and (3) Impact on workers’ health.

The “Restructuring the work teams” category includes adjustments faced by the professionals to adapt the work teams. The workers interviewed revealed experiences with greater impact regarding distancing/reallocation of the professionals belonging to the risk group who had to leave their work groups and places in order to assemble the COVID-19 units, as well as regarding establishment of new routines and protocols. In the non-COVID units, concern was evidenced about PPE availability and the constant changes in the care flows in order to maintain distancing of uncontaminated patients from the suspected cases, which was a generator of many uncertainties due to the care rules.

*[...] for us it was all uncertain, we didn’t know if we’d have the staff reallocated to other areas and if the patients would continue to be cared here with us [...] it was a very volatile scenario [...] it was all very unknown and the guidelines were changing every day. [...] it demanded a lot from our team [...]* (NUR 2 - Non-COVID-19).

*At first we had a lot of suffering related to all these changes, of so many people, of a complete restructuring in the service, this change was very intense, it required an adaptation like never before in our life.* (NUR 3 - COVID-19).

*[...] enough people with temporary contracts were hired to work in the COVID units, because there were a lot of sick leaves. [...] these units were given differentiated attention [...] the colleagues who replace us [sick leaves] are normally the same [...] the technicians when they came to help us, also had experience in isolation, so this made it all much easier because they were more used to the routines.* (NUR 5 - COVID-19).

The “Understanding risk and work demands” category addressed the professionals’ perception in view of the increased work demands, with experiences more emphasized by the workers of the COVID-19 units, who were subjected to increased complexity of the work tasks, as well as described themselves as victims of prejudice within the institution. The fear of exposure to the virus and the disease was present in the statements of the interviewees, especially those from the non-COVID-19 units, as they felt less privileged in PPE distribution by the institution.

*[...] when me and my colleagues from the ICC (Intensive Care Center) came down to change clothes in the locker room, peers from the other units screamed and told us to go away, because we weren’t allowed to change clothes in the same place. Even though we were exhausted and with no protection equipment and sanitized [...] this moment was very shocking for us, because they didn’t want to get in the elevators with us.* (NUR 3 - COVID-19).

*Too afraid of having the disease, of taking it home, [fear of caring] when the patient arrives with a suspicion, or knowing that such peer died and that I had worked with him. Another one [colleague] who had children of the same age as mine, died, another two hospital peers died, about 10 people from the institution died, I said “my God in Heaven”. In late March and April there was one death after another, a horror. I’m powerless, panicked and people are dying around me.* (NUR 9 - Non-COVID-19).

In the “Impact on workers’ health” category, the professionals talked about the symptoms experienced and/or worsening in the pre-existing clinical conditions due to their performance in the pandemic. Anxiety and depressive symptoms, insomnia and their somatization were the most found in the statements of the participants interviewed.

*[...] everything had to be hastily remanaged and restructured because of the situation and because the hospital is a reference for the care of COVID-19 patients [...] Walking into the hospital and seeing your peers not getting in the same elevator. So it was panic. Afraid of putting on the PPE in the wrong way before entering the room, of becoming contaminated, panic before leaving the room. [...] for me it was difficult to see my peers suffering [...] no one is ready to experience that [...] I haven’t stopped crying, I’m still recovering from last week. I have an appointment now, maybe I have to start with a medication, because I’m not getting it, it’s being too much for me, just to think that I’m going back to COVID. I think that the people who are working on the front line, they’re all going to get emotional sequelae, there’s no way they’re not getting them.* (NT 5 - COVID-19).

*I had enough contact with a lot of COVID patients without any warning. It also causes a little anxiety. I was afraid I had contracted COVID because I had a headache three days in a row, took medication and was no good, also tachycardia and shortness of breath. I was scared to death “I’m going to put an oximeter on to see if I’m saturating well” because I was thinking “my God, I’m going to die.” But I think it was tension, pure tension.* (NT 2 - Non-COVID-19).

The interviews expressed many feelings and symptoms of distress and illness, both in the professionals who worked in the COVID-19 and non-COVID-19 units. However, the relationship of these reports with the work experiences showed that the triggering factors did not have the same similarities, as professionals from the COVID-19 units had their causes in death, in the suffering of patients who did not respond to the treatment and in the urgency for new learning and definitions of care processes. In the non-COVID-19 units, the reports about health repercussions were related to the fear of having to deal with suspected COVID-19 patients or even approaching colleagues who worked directly with infected patients. The perception that PPE availability was different in the performance areas was the justification mentioned for these professionals’ fear.

Figure 2 shows the integration of the “QUAN” and “qual” results, combined by data connection and distributed across the units devoted and not devoted to the care of COVID-19 patients.

**Figure 2:** Joint-display of the connection between the QUAN (n=845) and qual (n=19) results about the implications of the Nursing team’s performance in units devoted and not devoted to the care of COVID-19 patients. Rio Grande do Sul, RS, Brazil, 2020-2021

QUAN RESULTS	qual RESULTS	
*QUAN - Socio-occupational profile*	*qual - Category 1: Restructuring the work teams*	
Mixed result: There are differences in composition of the Nursing teams working in COVID-19 and non-COVID-19 units		
	COVID-19 units	Non-COVID-19 units
Male workers (p=0.004), of black/brown/other skin color (p=0.015), with temporary contracts (p<0.001), with shorter working time in the area (p<0.001), in the institution (p<0.001) and in the sector (p<0.001) and those who had another job (p<0.001) prevailed in the COVID-19 units.	*The other issue was that it changed a lot, from my peers who worked with me in the afternoon, we have none, are all new and there are many colleagues with temporary contracts, emergency, from other units, our leaders [...]* (NT 1 - COVID-19).	[...] they kept treating all of them [patients], but with many restrictions, so it required a lot of our team [...] we needed to make several adaptations [...] peers got sick and we had to reorganize our work schedule, the flow of patients too [...] (NUR 2 - Non-COVID-19).
Workers with chronic diseases (p<0.001) and self-declared as belonging to the risk group (p<0.001) prevailed in non-COVID-19 units	*It was very difficult when I got there, employees who were considered risk group were relocated to other units, so some employees who were from the unit had to leave and to cover these employees they had to bring people from other units, like me [...] they directed people who were from the risk group, aged individuals, pregnant women, lactating and I went to the COVID-19 unit.* (NT 5 - COVID-19).	We were with enough patients from another area [unit specialty]. We were working with reduced staff, we should have been six professionals, but we actually were five, sometimes we happened to be four one night, four people taking care of the whole unit and complying with the workload. (NT 2 - Non-COVID-19).
*QUAN - Risk perception and work demands*	*qual - Category 2: Understanding risk and work demands*	
Mixed result: There are differences in composition of the Nursing teams working in COVID-19 and non-COVID-19 units		
	COVID-19 Units	Non-COVID-19 units
The increase in work requirements during the pandemic (p<0.001) was higher among workers from the COVID-19 units.	*[...] in addition to taking care of the comorbidities of each patient, we had some who were admitted to the hospital due to clinical problems, postoperative patients, all together, plus decompensation due to the COVID disease; so we had to adapt a lot, seek knowledge about what we could do [...]* (NT 10 - COVID-19).	*After a couple of days that he came out of the surgery, he tested positive and the whole team was contaminated, only the anesthetists didn’t because they were taking more care, with face shield and everything. How can you possibly deal with this situation? It’s all very new, there’s no treatment and when we least expect the patient has COVID.* (NT 3 - Non-COVID-19).
Workers from non-COVID-19 units experienced greater fear of exposure to the risk of contamination (p=0.028).	*I was always very tired, doing a lot of 12h shifts, morning and afternoon or afternoon and evening. And it’s very tiring, mental health is extreme, it’s not just doing my job knowing that I’m risking myself, that’s all. I’m going to work knowing that I have to do everything thoroughly, paramentation and de-paramentation and the things inside the room, so as not to get contaminated, then I start to get nervous, I enter the room with a very apprehensive emotional load, I can’t enter calm.* (NT 4 - COVID-19).	*At first it was very difficult, I remember a patient I took care of, a gentleman who had conjunctivitis, quite a lot of eye secretion and we were without PPE. I did eye hygiene, I was very close to him, then on the other day: COVID! The patient in front also tested positive and died after a few days. I stood there pondering: ‘my Lord! where am I, my Lord?!’ After a week in contact with the patient we would find out it was COVID and we isolated the whole room, we switched all the patients to another sector, took to isolation, it was a mess, my God in Heaven and us in the middle. Because we feel lost, because it’s us caring for people, and who cares for us? Nobody. It took a long time to have some care for ourselves, at least where I was, to provide PPE, but this equipment was rationed.* (NT 2 - Non-COVID-19).
	*[...] my last week was about having to choose who’s going to die and we didn’t study for that. I try, I’m very shaken by this last week [crying] and I’m really glad I’m on vacation because no one is really ready to deal with these situations. This week we had 4 patients and they chose me and the other 3 knew that they hadn’t been chosen and we were on the desperate side, seeing them, young people, but with no respirator and no ICU [Intensive Care Unit] bed. (NT 5 - COVID-19).*	
*QUAN - Implications of the pandemic on workers’ health*	*qual - Category 3: Impact on workers’ health*	
Mixed result: The pandemic similarly impacted the health of Nursing workers working in COVID-19 non-COVID units		
	COVID-19 Units	Non-COVID-19 units
Starting to use medication during the pandemic (p=0.340), absence from work due to health reasons during the pandemic (p=0.218), absence from work due to suspected COVID-19 (p=0.351) and to COVID-19 diagnosis (p=0.221), self-assessment of the impact on physical health (p=0.597) and Minor Psychological Disorders (p=0.535) were also present among the workers who worked in COVID-19 and non-COVID-19 units.	*We witnessed scenes of anxiety and panic attacks in professionals, blackouts, paralysis and this will never leave our memory. They were extremely shocking moments because we saw these behaviors in old professionals, safe, capable, experts in the area. [...] and these scenes of fear, paralysis and panic still come into our heads. [...] at first it brought about negative impacts, because feelings, frustrations, fear, anxiety and a sensation of impotence were arising. [...] there was enough somatization, fear, anxiety. Anxiety crises, sometimes veiled.* (NUR 3 - COVID-19).	*The first patient I received that was positive made me feel afraid, that week I was very anxious, until 10 days I was very anxious. Thinking that I ended up touching the patient, and not having the proper protection.* (NUR 1 - Non-COVID-19).
	*It was very distressing for all of us. I was very anxious, I noticed that I had a kind of panic syndrome, because it was a lot of information, it was a lot of change. They had shifts where every night we took a patient to the ICU, so it generates a lot of anxiety in the group. [...] And that anxiety, that fear, a lot of not knowing what’s going to happen anyway. I thought it affected a lot, I was seeing that I had panic syndrome. And my peers were feeling that way, too.* (NUR 5 - COVID-19).	*Headache, in these last months I have it almost every day, I had gastrointestinal problem due to stress, anxiety and I couldn’t sleep at night, I woke up at 2 am thinking “I will have to work”, I woke up and the headache started. [...] I’m very exhausted, very tired, because there’s a lot of post COVID patients and they are all bedridden and overload us. [...] it’s a sum of the burden of the pandemic, of having family members, having a life outside this place, having problems. My husband got out of isolation last week, he had COVID, my young son had bronchitis and I get all that pressure thinking “am I bringing COVID home?”, I’m terrified of contaminating my son. I live under this pressure.* (NT 8 - Non-COVID-19).

## Discussion

Composition of the teams that worked in the COVID-19-and non-COVID-19 units revealed a number of differences, even with regard to sex. Although Nursing is a mostly and historically female profession, both in this study and in another one carried out during the pandemic, there was a significant number of male professionals comprising the teams of units devoted to the care of patients infected with COVID-19^32^. This fact can be due to the role socially attributed to men in the culture, linked to virility and courage, which often consolidates denial of vulnerability and fear^33^. On the other hand, the result found can be related to the female attribute of household activities, with responsibilities for the care of frail individuals such as older adults and children^34^, thus preferring to work in non-COVID-19 areas.

In addition, mixed race, black and others was prevalent in the composition of teams from COVID-19 units, as well as temporary employment contracts in the institution, more than one job and less time of experience in the profession, in the institution and in the unit. Afro-American people represent a significant number of workers in Nursing and their predominance in COVID-19 units can be understood in view of the temporary employment contracts and their relationship with unemployment and the double working hours already described in the literature, vulnerabilities that were increased in the pandemic for the less favored groups^35^.

Many changes were necessary and inevitable in order to prepare health services to face the pandemic. The expansion of beds for the creation of new sectors, the increase of night teams in the COVID-19 units due to the patients’ severity and the relocations of professionals that belonged to the risk group resulted in many emergency hires and also in dissolution of teams. Breaking of the work teams’ bonds to the detriment of the new dimensioning and distribution of professionals was mentioned by the interviewees as a difficult moment, which may have affected the social support that already existed in the work groups, requiring the construction of new bonds and work organization with new colleagues, even causing psychological illness^36^ ^-^ ^37^.

The training of new colleagues, sometimes recent graduates or without experience in hospital and/or intensive care, was reported by the interviewees of this study as an increase in workload. A number of studies have disclosed special attention to inexperienced nurses, who had fewer skills to deal with work-related difficulties, as revealed by a Chinese study on psychological manifestations of decreased appetite, fatigue, insomnia, nervousness, frequent crying and even suicidal thoughts in inexperienced professionals who worked in COVID-19 units^10^ ^,^ ^38^.

A study that disclosed greater severity in the development of trauma secondary to the pandemic in nurses working in the units that did not deal directly with patients infected with COVID-19 suggests that this was due to the fact that the professionals from COVID-19 units volunteered for this service because they were more experienced^39^. However, this result differs from the current research, considering that the professionals from COVID-19 units had less experience in the profession, in the institution and also in the unit.

This study identified that the inexperience of the Nursing professionals who worked in the COVID-19 units represented an additional burden for the experienced ones, as they had to take on training and supervision to perform their duties. A number of studies were conducted on this support to inexperienced workers and nurses from Turkey pointed out the following among the difficulties dealing with the new working conditions: concern with possible errors by new colleagues, referring to the experience of anxiety by those who were already in the service by assuming adaptation of these professionals to the new sector^40^ ^-^ ^41^.

This constant concern for the possibility of incorrect actions by colleagues possibly ends up burdening the work of the most experienced, as there was an increase in work demands in the COVID-19 units^10^ ^,^ ^42^ also due to the rigor in PPE use, such as paramentation and de-paramentation and its prolonged use, as well as the change in the profile of the patients, who were distributed by specialties across the units, such as Oncology and Hematology, and had their particularities added to the coronavirus infection^10^ ^,^ ^43^.

The change in the patient profile was due to the need to create units devoted to the care of patients infected with the coronavirus, in order to control its dissemination. However, in addition to also manifesting severe forms of the disease, becoming critical care patients^38^, people affected by the virus also had other diseases such as cancer or diabetes, patients who had undergone surgeries, in the terminal phase of life and a wide variety of other comorbidities and situations, with the need for the professionals who were in these dedicated units to be trained to treat them^43^.

Another difficulty faced by the health services and that put Nursing professionals at risk was lack of PPE, which was a worldwide problem at the beginning of the pandemic, as well as the lack of training for its proper and prolonged use^43^ ^-^ ^44^, which caused pressure injuries^45^. In Italy, nurses in COVID-19 units linked these situations to the large number of contamination cases in the professionals, requiring the development of several protocols such as training for the proper use of PPE^46^.

For the professionals who were not working directly with infected patients, lack of adequate protection and the uncertainty of the fact that a given patient was not infected with coronavirus generated greater fear in the face of exposure to the risk for contamination during work. Diverging from this result, studies carried out with nurses showed greater fear felt by those who worked directly with infected patients, who also felt more unprotected^34^ ^,^ ^47^. Non-protection of health professionals, such as PPE scarcity, misuse and inadequacy, generated fear in those who worked during the pandemic, regardless of the performance unit.

The fear felt by the professionals of both types of unit in relation to being contaminated and transmitting the virus to their family members and friends was linked to the experiences of prejudice suffered by the professionals of the non-COVID-19 units, practiced by people in social spaces, who showed fear even if the protocols regarding distancing and use of masks were respected^48^. During the pandemic, stigmatization and prejudice brought about concerns to Nursing professionals, who sometimes face problems regarding the family and social support network that can negatively affect their mental health^15^ ^,^ ^48^.

On the other hand, the professionals from COVID-19 units had the same feeling, but in relation to the colleagues of the institution who did not work directly with infected patients, when they shared changing rooms, corridors and elevators, even when they were no longer wearing contaminated uniforms. Both felt the need to isolate themselves from people in general life in order not to be held responsible for contaminating anybody The professionals who dealt directly with COVID-19 also distanced from colleagues from other sectors, both because of the prejudice suffered and because they were not considered contamination means within the institutions^43^ ^,^ ^49^.

There were no statistically significant differences in sick leave, impact on physical health and suspicion of MPDs among Nursing professionals in COVID-19 and non-COVID-19 units and this differs from the findings of studies carried out with nurses from China, in which the professionals who did not work directly with infected patients presented higher levels of professional illness when compared to those who were in the COVID-19 ward^23^.

Among the possible explanations provided by the authors^23^, there is the fact that the professionals who worked directly with patients infected with COVID-19 feel better able to control the situation. This corroborates the current study, as the workers from the non-COVID-19 units reported not having felt prioritized to the detriment of the established flows and the materials provided at the first moment of the pandemic, generating a sensation of helplessness and non-protection, as they also ended up dealing with infected patients and also unsuspectingly, because many infected people were asymptomatic, which can increase exposure to the risk of infection and the fear felt^43^.

Regarding the finding about the feeling of fear in the face of the contamination risk experienced with higher prevalence by the professionals from non-COVID-19 areas, it is worth relating this to the study^50^ found more contaminated professionals in non-COVID-19 areas when compared to professionals from COVID-19 areas. In addition to weighing the negative experience of the feeling of fear found in the current study, a number of authors^12^ ^,^ ^51^ showed that the fear of being contaminated was a predictive factor for the development of depression among Nursing workers.

The studies developed with nurses showed higher levels of illness among those who work directly with infected patients^12^ ^,^ ^15^ ^,^ ^34^ ^,^ ^52^. In China^15^, they showed more severe degrees of depression, anxiety, insomnia and anguish; in Germany^12^ they had more depression, exhaustion and stress, when compared with professionals from the non-COVID-19 units, as well as in Iran^34^, where exhaustion and stress prevailed in these professionals. In the current study there was no difference regarding the psychological health of the professionals from different areas.

Although this study did not identify differences between the performance units with regard to MPD, their high prevalence among the participants stands out, as well as in a study carried out during the pandemic in Brazil^53^ with 490 professionals from a Nursing team, revealing that 30.4% of the participants had some mental disorder diagnosed, with moderately severe or severe anxiety as the most prevalent diagnosis, followed by moderately severe or severe depression. In Canada^54^, more than 50% of the participants in a study presented depression symptoms, with 42% moderate, serious or severe and more than 65% of the professionals had anxiety, with 22% serious or severe.

This study was developed entirely online, which may be one of the largest limitations to be considered, as the demands for remote interactions began to overwhelm the professionals in the pandemic, who often were not willing to stay in front of the computer or other digital medium for this reason, as well as the fact that the quality of the interviews was impacted by virtuality. The fact that the research was developed by nurses, who somehow also worked during the pandemic, facilitated the study logistics but generated negative emotions due to the impact of the pandemic on all life instances.

Another limitation was found in relation to time, a factor inherent to cross-sectional research, as it is a clipping of a given moment and resulting from the constant changes in the pandemic, such as daily changes in institutional flows, illness of professionals, increase in the number of contaminated people and individuals in need of care, which makes these results reflect a certain period of the pandemic.

This research contributed to the advancement of scientific knowledge, as it portrays a reality that was unexpected by Nursing and society. The results provided knowledge for health managers about the situations experienced by Nursing professionals and the need for improvements in their health and working conditions.

## Conclusion

The integrated data analysis allowed concluding that Nursing professionals who worked in COVID-19 and non-COVID-19 units suffered harms to health to the same extent, although with different occupational exposure regarding the requirements of work pace and complexity in COVID-19 units and the fear of contamination in non-COVID-19 units. Therefore, the hypothesis that the health impacts of the pandemic had affected professionals from different sectors of the Nursing practice was proved in this study.

There is an emphasis on the urgent need for improvements in the working conditions, in support and reception of the mental health demands of the professionals, who work/worked in coping with the pandemic, both in COVID-19 and in non-COVID-19 units. Measures to promote health and prevent illness and/or the complications of the distress already established should envision professionals from different Nursing care areas, considering the vulnerability to the coronavirus infection, as well as the psychosocial implications of working during the pandemic.
